# A cross sectional study of maternal ‘near-miss’ cases in major public hospitals in Egypt, Lebanon, Palestine and Syria

**DOI:** 10.1186/s12884-015-0733-7

**Published:** 2015-11-13

**Authors:** Hyam Bashour, Ghada Saad-Haddad, Jocelyn DeJong, Mohammed Cherine Ramadan, Sahar Hassan, Miral Breebaart, Laura Wick, Nevine Hassanein, Mayada Kharouf

**Affiliations:** Faculty of Medicine, Damascus University, Damascus, Syria; Faculty of Health Sciences, American University of Beirut, Beirut, Lebanon; Obstetrics and Gynecology department, Al Galaa Maternity Teaching Hospital, Cairo, Egypt; Institute of Community and Public Health, Birzeit University, Birzeit, Palestine; Social Research Center, American University in Cairo, Cairo, Egypt; Institute of Community and Public Health, Birzeit University, Ramallah, Palestine; Independent, Reproductive Health Consultant, Cairo, Egypt

**Keywords:** Maternal near miss, Severe maternal morbidity, Middle-Eastern countries, WHO near miss tool, Referral hospitals

## Abstract

**Background:**

The maternal near-miss approach has been increasingly used as a tool to evaluate and improve the quality of care in maternal health. We report findings from the formative stage of a World Health Organization (WHO) funded implementation research study that was undertaken to collect primary data at the facility level on the prevalence, characteristics, and management of maternal near-miss cases in four major public referral hospitals - one each in Egypt, Lebanon, Palestine and Syria.

**Methods:**

We conducted a cross sectional study of maternal near-miss cases in the four contexts beginning in 2011, where we collected data on severe maternal morbidity in the four study hospitals, using the WHO form (Individual Form HRP A65661). In each hospital, a research team including trained hospital healthcare providers carried out the data collection.

**Results:**

A total of 9,063 live birth deliveries were reported during the data collection period across the four settings, with a total of 77 cases of severe maternal outcomes (71 maternal near-miss cases and 6 maternal deaths). Higher indices for the maternal mortality index were found in both Al Galaa hospital, in Egypt (8.6 %) and Dar Al Tawleed hospital in Syria (14.3 %), being large referral hospitals, compared to Ramallah hospital in Palestine and Rafik Hariri University hospital in Lebanon. Compared to the WHO’s Multicountry Survey using the same data collection tool, our study’s mortality indices are higher than the index of 5.6 % among countries with a moderate maternal mortality ratio in the WHO Survey. Overall, haemorrhage-related complications were the most frequent conditions among maternal near-miss cases across the four study hospitals. In all hospitals, coagulation dysfunctions (76.1 %) were the most prevalent dysfunction among maternal near-miss cases, followed by cardiovascular dysfunctions. The coverage of key evidence-based interventions among women experiencing a near-miss was either universal or very high in the study hospitals.

**Conclusions:**

Findings from this formative stage confirmed the need for quality improvement interventions. The high reported coverage of the main clinical interventions in the study hospitals would appear to be in contradiction with the above findings as the level of coverage of key evidence-based interventions was high.

**Electronic supplementary material:**

The online version of this article (doi:10.1186/s12884-015-0733-7) contains supplementary material, which is available to authorized users.

## Introduction

The maternal near miss approach has been increasingly used as a tool to evaluate and improve the quality of care in maternal health [1, 2].A Maternal Near Miss (MNM) is defined by the World Health Organization (WHO) as a woman who nearly died but survived a complication that occurred during pregnancy, childbirth or within 42 days of termination of pregnancy [1].

Despite increased interest in the near miss concept, the measurement of maternal near-miss cases and severe maternal outcomes remained non-standardized [3] and therefore WHO undertook a collaborative international process to establish internationally agreed criteria for near-miss cases [4–7]. Over the last decade, an increasing volume of research has been carried out with the aim of identifying maternal near-miss cases in order to understand health system failures in relation to obstetric care and to help determine solutions to address them [7].WHO’s own multicountry survey (MCS) of near-miss cases in 359 hospitals located in 29 countries, detailed analysis of which is included in a special supplement of the British Journal of Obstetrics and Gynaecology, provides a great deal of understanding about the number, severity and associated obstetric factors of near-miss cases in those settings, the personal characteristics of women who experience severe morbidity, as well as the types of interventions associated with those cases in the contributing health facilities [6]. The link between maternal complications and perinatal survival is well-documented based on the principle of the continuum of pregnancy, labour, and the postpartum period [8].

In the Middle East region, however, there have been few studies measuring near-miss cases and their characteristics. Although hospitals in Jordan, Lebanon, Palestine and Qatar [6] were included in the WHO MCS study, specific results from Middle Eastern countries have not been published separately. A study in urban Iraq, using the WHO near miss definitions, found the prevalence of maternal deaths and near-miss cases to be relatively low [9].

Since the late 1990s, an established research network entitled the Choices and Challenges in Changing Childbirth^1^ has been conducting research on maternal health in the Arab region. The study being reported here is an implementation research project undertaken by members of that network.Thef ourArab contexts included in this research; namely Egypt, Lebanon, Palestine and Syria have reported good progress in maternal health indicators [10]. These countries, inspite of their great diversity, share certain commonalities in terms of maternal health and health systems. In the four settings, there has been an increase in facility-based births and maternal mortality has decreased. Maternity care is largely provided by obstetricians (with only a minor role for midwives, except in Palestine where they assist the normal births in public hospitals), and in the case of Lebanon through a dominant private sector [11], while in Egypt and Syria predominantly through the public sector but with a growing private sector [12].The Palestinian Authority provides childbirth care in governmental hospitals at low cost to the population (including Palestinian refugees), alongside a growing private and NGO sector. There is rigorous evidence generated by our research network and others, however, that the quality of maternal health care needs improvement in the four settings. For example, routine practices relating to maternal and neonatal health have been found to deviate from safe and evidence-based practices [13]. Excessive levels of medical intervention such as cesarean-section exist [14], with national hospital rates of c-section reaching as high as 46 % in Lebanon, for example [14]. Labour companionship is largely prohibited in public hospitals in Egypt, Palestine and Syria, although it is increasingly permitted in Lebanon [13]. Previous studies by our research network have also identified problems of substandard obstetric care such as inappropriate routine administration of oxytocin for labour augmentation [15, 16]. Furthermore, insufficient identification and management of high-risk cases, referral and reportingas well as inadequate systems were found to be problematic and major contributors to maternal mortality in these settings. In Egypt, for example, a national confidential inquiry found that 43 % of avoidable factors contributing to maternal mortality were due to substandard care by the obstetric team [17, 18]. Similarly in Syria, 74 % of maternal deaths were deemed by committees of physician experts to be preventable [19].

In this paper we report on the prevalence of maternal near-miss cases in the four major public hospitals in Egypt, Lebanon, Palestine and Syria during the formative stage of a larger study that was designed to test the acceptability, feasibility and effectiveness of a multifaceted strategy combining clinical audit, feedback and engaging opinion leaders to improve quality of care in the four major public hospitals. Proper management of maternal near miss was used as the main outcome of the above mentioned WHO-funded implementation research .We also assess here the pregnancy and delivery complications and their contributing factors, as well as the socio-demographic factors related to severe outcomes of pregnancy.

## Methods

We conducted a cross-sectional study of maternal near-miss cases in four public hospitals in the four settings beginning in 2011. A cross sectional study of severe maternal morbidity was carried out using the WHO form for identifying near-miss cases (Individual Form HRP A65661- Additional file 1) as in the multicountry survey on maternal and newborn health [6]. Eligible women included all women giving birth at those hospitals or referred to those hospitals during the data collection period and all women who had a severe maternal outcome (a near-miss case or maternal death) irrespective of the gestational age and delivery status, up to the seventh postpartum day. This data collection tool was used to identify women with complications and those severe cases which correspond to the near miss criteria (defined as women with organ dysfunction related to pregnancy or childbirth, including abortion and ectopic pregnancy).

### Study settings

The study was carried out in four major hospitals in the capital cities of Egypt, Lebanon, and Syria. In Palestine, it was carried out in the city of Ramallah in the West Bank. The purposive selection of the four hospitals was based on their characteristics as large referral teaching hospitals in the public sector with qualified health professionals and high case loads. The Egyptian hospital is the main referral and teaching tertiary hospital in Cairo; the Lebanese hospital is the only public maternity hospital in Beirut; the Palestinian hospital is a main public referral hospital serving West Bank Palestinians; and the Syrian hospital is the largest teaching maternity hospital in the country. The previous experience of our research network with these hospitals was also an advantage.

The four hospitals are, respectively, affiliated to the Ministry of Health in Egypt (Al Galaa hospital), Lebanon (Rafik Hariri University Hospital - RHUH), and Palestine (Ramallah hospital) but the fourth (Dar Al Tawleed University hospital) in Damascus is affiliated to the Ministry of Higher Education. At the time of data collection, the average number of deliveries per year was very high in both the Syrian and Egyptian hospitals (15,000 and up to 25,000, respectively), over 4,000 in Ramallah hospital and less than 1,500 in RHUH in Beirut.

All the hospitals’ contexts were affected by the political events that took place in the Arab countries beginning in 2011 (and earlier in Palestine and Lebanon). It is not the aim of this paper to describe those changes and their effects, which were most pronounced during the subsequent intervention stage of this study; a separate paper related to this is under preparation.

### Data collection

In each hospital, a research team was responsible for carrying out the data collection in a prospective manner. During the data collection period, all women admitted to the four health facilities were screened according to the inclusion criteria by the research team. Data collectors (research assistants, midwives and resident doctors trained by the research team) undertook a daily review of all admissions to labour, delivery, postpartum, emergency or intensive-care units to identify all eligible women, using the WHO Individual form for the Multicountry Survey on Maternal and Neonatal Health. An Arabic version of the form was developed by translation and back-translation for validation (Additional file 2). The Manual of Operations of the WHO MCS (including all operational definitions) guided the data collection in this study [20] and the team developed its own operational manual contextualized to the study sites. Data collection periods varied across the settings according to hospital delivery load, with Egypt, Syria and Palestine having relatively similar data collection period (12–14 weeks) while Lebanon had a longer period (41 weeks) due to the low case load compared to the other three hospitals. As the medical records in the hospitals in Egypt, Palestine and Syria were not available electronically, unlike those in Lebanon, the data collection process was more laborious. High case loads mainly in Egypt and Syria posed additional challenges. Nevertheless, data collection was standardized across the contexts and challenges were discussed through six regional meetings of the research teams for close coordination among them. To further ensure data quality and standardization, several procedures were applied in each site, such as thorough training of the data collectors, quick checks of the completed form by the researchers prior to data entry, incorporating conditions and queries within the data entry program (for example, date of discharge not more than 7 days from date of delivery or number of previous births not to exceed the number of pregnancies and so on) and random checks on entered data as well as a double entry on 10 % of the forms.

### Data analysis

Data collected by the four research teams were entered after data cleaning using the public domain CSPro software.^2^^2^ The data entry manual was developed by a statistician from the research team and shared with the country teams. The data analysis template was then shared after a research team meeting at the coordinating institution (Faculty of Health Sciences of the American University of Beirut) and thus data analysis was carried out by the four country teams (using SPSS version 21) and then compiled and discussed in a large group. Frequencies of maternal near miss events and maternal deaths were generated and main maternal near miss indicators were calculated accordingly. The socio-demographic characteristics and obstetric profiles of all women and women who experienced an MNM event were presented through cross-tabulation. Additionally, statistical significance was tested, using the Chi square test or the Fischer test (when the number of cases was lower than five), to compare characteristics and profiles of women who experienced a near miss event compared to women who did not have an near miss event. Frequencies of the contributing conditions and types of organ dysfunction among the maternal near-miss cases were presented as a proportion of the number of maternal near-miss cases by country. The coverage of key clinical interventions, in the four countries, was examined as a proportion of those who received the intervention per the target population.

### Ethical considerations

The study protocol was reviewed by the WHO specialist panel as well as approved by the WHO Research Ethics Review Committee. The study was approved by a local ethical review committee at the university level in Palestine and Syria and at the hospital level in Egypt, and then by the Institutional Review Board (IRB) of the American University of Beirut as the coordinating institution. Written consents from individual participants (female patients at the hospitals) were exempted by the IRB at the Amercian Univeristy of Beirut and also by the Ethics Research Commiittee at the World Health Organizaion, as data collection for this stage of the study relied solely on extraction of data from medical records by trained hospital staff. Hospital staff who extracted data were however required to undertake an online training in research ethics and certificates were shared with the IRB. All data collected was immediately coded and entered for analysis by the research team. Data forms are kept locked in the coordinators’ offices and on password-protected computers of the research team members.

## Results

Table 1 compares the maternal health indicators (severe maternal morbidities) in the four hospitals in the respective contexts.Out of 9,063 deliveries of live births, a total of 77 cases of severe maternal outcomes (71 maternal near miss cases and 6 maternal deaths) were reported through the data collection period in the four settings. Higher levels of the maternal mortality index were identified in both Al Galaa hospital in Cairo (8.6 %) and Dar Al Tawleed Hospital in Damascus (14.3 %). This index measures the quality of care at the facility and a higher index suggests that more women with life-threatening conditions die, i.e. lower quality of care, whereas a lower index shows that fewer women with life-threatening conditions die, hence better quality of care [20].

**Table 1:** Maternal health indicators in the study hospitals in Egypt, Lebanon, Palestine and Syria as compared to findings from the WHO MCS Study

Country	Data collection period (2012–2013)	Live births (LB)	Maternal deaths	Near-miss cases	SMOR per 1000 LB^c^	MNMR per 1000 LB^d^	MNM mortality ratio^e^	MI (%)^f^
Egypt	12 weeks	2641	3	32	13.3	12.1	11:1	8.6
Lebanon	41 weeks^a^	1171	0	5	4.3	4.3	5:0	0.0
Palestine	14.5 weeks	1244	0	16	12.9	12.9	16:0	0.0
Syria	12 weeks	4007	3	18	5.2	4.5	6:1	14.3
All		9063	6	71	8.5	7.8	11.8:1	7.8
*Overall WHO MCS results (N = 29)* ^b^		*306,771*	*486*	*2538*	*9.9*	*8.3*	*5.2:1*	*16.1*
*WHO MCS–Countries with moderate MMR(N = 15)* ^b^		*134,545*	*49*	*824*	*6.5*	*6.1*	*16.8:1*	*5.6*

^a^Due to the low average annual number of deliveries in RHUH, the team decided to continue data collection for a longer period of time than the other study sites

^b^Source [21]:

^c^SMOR - Severe Maternal Outcome Ratio: the number of women with life-threatening conditions (MNM + MD) per 1000 live births (LB). This indicator gives an estimate of the amount of care and resources that would be needed in an area or facility (SMOR = MNM + MD/LB) [1]

^d^MNMR-MNM Ratio: the number of maternal near-miss cases per 1000 live births (MNMR = MNM/LB). Similarly to the SMOR, this indicator gives an estimation of the amount of care and resources that would be needed in an area or facility [1]

^e^Maternal near-miss mortality ratio (MNM: 1 MD):the ratio between maternal near-miss cases and maternal deaths. Higher ratios indicate better care [1]

^f^[Maternal] Mortality Index (MI): the number of maternal deaths divided by the number of women with life-threatening conditions expressed as a percentage (MI = MD/MNM + MD). The higher the index the more women with life-threatening conditions die (low quality of care), whereas the lower the index the fewer the women with life-threatening conditions die (better quality of care) [1]

**Fig. 1:**
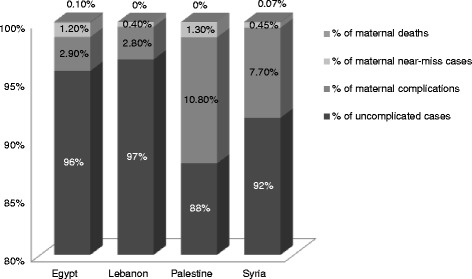
Distribution of maternal complications (excluding maternal near-miss & maternal death cases), near-miss cases and maternal deaths across the four study hospitals

Figure 1 shows the continuum of severity of maternal outcomes in terms of proportions of maternal complications, maternal near-miss cases and maternal deaths in the four study hospitals during the study period. The highest proportion of complications was found in both the Ramallah hospital in Palestine (10.8 %) and Dar Al Tawleed hospital in Damascus, Syria (7.7 %). Lower proportions were noted in Al Galaa hospital in Cairo, Egypt and RHUH in Beirut, Lebanon (less than 3 % in both hospitals). The proportion of maternal near-miss cases was higher than 1 % in Egypt’s Al Galaa hospital and in Palestine’s Ramallah hospital.

The socio-demographic characteristics of all women and women who experienced a near-miss eventare shown in Table 2 (with the exception of Egypt where, given the very high caseload, it was not possible to collect data on all women). Women who experienced near-miss events were significantly older in both Palestine and Syria (*p* < 0.000) and were less educated in Lebanon (*P* = 0.015) than those who did not experience near-miss events. Only in Palestine, women who experienced a maternal near-miss case had significantly higher parity than their counterparts (*P* = 0.001).

**Table 2:** Socio-demographic characteristics of all women and women who experienced a near-miss eventin the four study hospitals

Hospital/Country	Egypt			Lebanon			Palestine			Syria		
	Al Galaa Hospital			Rafik Hariri University Hospital			Ramallah Hospital			Dar Al Tawleed Hospital		
	All women^b^	Women with an MNM	P-value	All women	Women with a MNM	P-value^a^	All women	Women with a MNM	P-value^a^	All women	Women with a MNM	P-value^a^
	N (%)	N (%)		N (%)	N (%)	N (%)	N (%)	N (%)	N (%)	N (%)	N (%)	
Age mean (SD) in Years	*(NA)*	27.8 (4.6)		27.7 (6.4)	30.6 (5.6)		27.6 (5.8)	32.4 (8.3)		25.8 (6.7)	28.7 (8.5)	0.045
Age			*(NA)*			0.718			0.000			0.000
<20 year	*(NA)*	0 (0.0)		95 (8.1)	0 (0.0)		115 (9.3)	0 (0.0)		465 (13.7)	2 (11.1)	
20-35	*(NA)*	31 (96.9)		915 (77.7)	4 (80.0)		992 (80.2)	8 (50.0)		2653 (78.2)	10 (55.6)	
36+	*(NA)*	1 (3.1)		168 (14.3)	1 (20.0)		130 (10.5)	8 (50.0)		275 (8.1)	6 (33.3)	
Marital Status			*(NA)*			0.873			0.974			0.010
Married	*(NA)*	30 (93.8)		1175 (99.7)	5 (100)		1234 (99.8)	16 (100)		3369(0.2)	17(100)	
Single/widow/divorced	*(NA)*	2 (6.2)		3 (0.3)	0 (0.0)		2 (0.2)	0 (0.0)		7 (0.2)	0 (0.0)	
Years of schooling^c^			*(NA)*			0.015			*(NA)*			0.244
No education	*(NA)*	*(NA)*		12 (1.0)	0 (0.0)		*(NA)*	*(NA)*		166(7.5)	1(10)	
Primary (1–6 years)	*(NA)*	*(NA)*		216 (18.3)	4 (80.0)		*(NA)*	*(NA)*		796(36)	5(50)	
Lower secondary (7–9 years)	*(NA)*	*(NA)*		537 (45.6)	0 (0.0)		*(NA)*	*(NA)*		829(37.4)	1 (10)	
Upper secondary (10–12)	*(NA)*	*(NA)*		289 (24.5)	1 (20.0)		*(NA)*	*(NA)*		290 (13.1)	3(30)	
Tertiary (>12 years)	*(NA)*	*(NA)*		124 (10.5)	0 (0.0)		*(NA)*	*(NA)*		133(6)	0 (0)	
Parity			*(NA)*			0.444			0.001			0.447
0	*(NA)*	6 (18.8)		378 (32.1)	3 (60.0)		261 (21.1)	1 (6.3)		826(24.1)	5 (27.8)	
1-2	*(NA)*	12 (37.5)		576 (48.9)	1 (20.0)		488 (39.5)	3 (18.8)		1519(44.4)	5 (27.8)	
3-4	*(NA)*	13 (40.7)		170 (14.5)	1 (20.0)		313 (25.3)	5 (31.3)		773 (22.6)	5 (27.8)	
5 +	*(NA)*	1 (3.1)		53 (4.5)	0 (0.0)		175 (14.1)	7 (43.8)		305(8.9)	3(16.2)	

^a^All P-values represent the difference between the two groups of women: MNM and women who are not MNM

^b^Given high case load at Al Galaa Hospital in Egypt, it was not possible to collect data on all women

^c^Years of schooling was not included in the medical records of Al Galaa Hospital

(NA) = Not Applicable

Table 3 shows the obstetric profile of all women (from Lebanon, Palestine and Syria) and near-miss cases in the four hospitals. There was a significant difference between women who experienced a maternal near-miss event and those who did not. More women who experienced near-miss events had a history of two previous c-sections in Palestine and a lower gestational age at delivery and more frequent reports of no labour onset as compared to women without maternal near miss in Lebanon, Palestine and Syria, with statistically significant differences. A statistically significant lower proportion of women with maternal near-miss event had a normal delivery as compared to women without maternal near-miss event in all contexts (*p* < 0.001).

**Table 3:** Obstetric profile among all women and women who experienced a near-miss event in the four study hospitals

	Egypt			Lebanon			Palestine			Syria		
	Al Galaa Hospital^b^			Rafik Hariri University Hospital			Ramallah Hospital			Dar Al Tawleed Hospital		
	All women^b^	MNM	P-value^a^	All women	MNM	P-value^a^	All women	MNM	P-value^a^	All women	MNM	P-value^a^
	N (%)	N (%)		N (%)	N (%)		N (%)	N (%)		N (%)	N (%)	
Previous C Section			*(NA)*			0.123			0.062			0.227
0	*(NA)*	16 (50.0)		909 (77.2)	3 (60.0)		1019 (82.4)	10 (62.5)		2793(81.6)	12 (66.7)	
1	*(NA)*	5 (15.6)		157 (13.3)	0 (0.0)		138 (11.2)	4 (25.0)		365 (10.7)	3 (16.7)	
2+	*(NA)*	11 (34.4)		112 (9.5)	2 (40.0)		80 (6.5)	2 (12.5)		265 (7.7)	3 (16.7)	
Gestational Age at delivery			*(NA)*			0.002			0.000			0.000
<33 weeks	*(NA)*	13 (43.3)		64 (5.4)	2 (40.0)		32 (2.6)	4 (26.7)		146 (4.3)	6 (33.3)	
33-36 weeks	*(NA)*	8 (26.7)		129 (11.0)	2 (40.0)		109 (8.8)	2 (13.3)		137 (4)	3 (16.7)	
37+ weeks	*(NA)*	9 (30.0)		985 (83.6)	1 (20.0)		1093 (88.6)	9 (60)		3138 (91.7)	9 (50.0)	
Onset of Labour			*(NA)*			0.043			0.000			0.000
Spontaneous	*(NA)*	3 (14.3)^c^		858 (72.8)	1 (20.0)		952 (77)	7 (43.8)		3154 (94.1)	9 (64.3)	
Induced	*(NA)*	3 (14.3)		238 (20.2)	3 (60.0)		131 (10.6)	2 (12.5)		18 (0.5)	1 (7.1)	
No Labour	*(NA)*	15 (71.4)		79 (6.7)	1 (20.0)		154 (12.4)	7 (43.8)		179 (5.3)	4 (28.6)	
Mode of delivery			*(NA)*			0.007			0.000			0.000
Vaginal Delivery	1248 (41.5)	3 (9.4)		746 (63.3)	0 (0.0)		927 (74.9)	5 (31.1)		2451 (71.6)	5 (27.8)	
Caesarean section	1488 (49.5)	21 (65.6)		432 (36.7)	5 (100)		307 (24.8)	8 (50.0)		913 (26.7)	11 (61.1)	
All Other	268 (8.9)	8 (25.0)		0 (0.0)	0 (0.0)		3 (0.3)	3 (18.9)		58(1.7)	2 (11.1)	

^a^All P-values represent the difference between the two groups of women: MNM and women who are not MNM

^b^Given high caseload at Al Galaa Hospital in Egypt, it was not possible to collect data on all women

^c^the total number of cases might not add exactly to the total number of maternal near-miss cases due to missing data

(NA) = Not Applicable

The contributing conditions as defined by WHO guidelines [20] among the maternal near-miss cases are shown in Table 4. Overall, haemorrhage-related complications were the most frequent conditions among maternal near-miss cases across the four study hospitals. At Al Galaa hospital in Egypt, placenta previa and ectopic pregnancy complications were the leading contributing conditions to maternal near-miss cases. At RHUH in Lebanon, similar to Al Galaa in Egypt, complications from placenta previa were frequent among maternal near-miss cases, in addition to accreta, increta, percreta placenta and hepatic disease conditions. In Ramallah hospital in Palestine, the leading contributor to maternal near-miss cases was postpartum haemorrhage followed by other types of obstetric haemorrhage. In Dar Al Tawleed hospital in Syria, the leading contributing conditions were placenta previa and postpartum haemorrhage. Among other contributing conditions, anemia was the main contributing factor in the four countries combined (15.5 % of all cases).

**Table 4:** Frequency of contributing conditions among maternal near-miss cases in the four study hospitals

	Egypt	Lebanon	Palestine	Syria	All
	Al Galaa Hospital	Rafik Hariri University Hospital	Ramallah Hospital	Dar Al Tawleed Hospital	
	(*N* = 32)	(*N* = 5)	(*N* = 16)	(*N* = 18)	(*N* = 71)
	N (%)	N (%)	N (%)	N (%)	N (%)
Haemorrhage					
Abortion related haemorrhage	1 (3.1)	0 (0)	0 (0)	2(11.1)	3 (4.2)
Ectopic pregnancy	7 (21.9)	0 (0)	1 (6.3)	0 (0)	8 (11.3)
Placenta praevia	11 (34.4)	2 (40.0)	0 (0)	4 (22.2)	17 (23.9)
Accreta/increta/percreta placenta	3 (9.4)	2 (40.0)	1 (6.3)	3 (16.7)	9 (12.7)
Abruptio placenta	3(9.4)	0 (0)	0 (0)	1 (5.6)	5 (7)
Ruptured uterus	3(9.4)	0 (0)	1 (6.3)	2 (11.1)	7 (9.9)
Postpartum haemorrhage	2(6.3)	1 (20.0)	6 (37.5)	4 (22.2)	15 (21.1)
Other obstetric haemorrhage	3(9.4)	0 (0)	5 (31.3)	1 (5.6)	7 (9.9)
Infection	0 (0)	0 (0)	0 (0)	0 (0)	0 (0)
Hypertension					
Chronic hypertension	1 (3.1)	0 (0)	1 (6.3)	1 (5.6)	3 (4.2)
Pre-eclampsia (excludes eclampsia)	1(3.1)	0 (0)	1 (6.3)	2 (11.1)	5 (7)
Eclampsia	2 (6.3)	1 (20.0)	1 (6.3)	1 (5.6)	3 (4.2)
Other conditions					
HIV +/AIDS/HIV wasting syndrome	0 (0)	0 (0)	0 (0)	0 (0)	0 (0)
Anaemia	1 (3.1)	1 (20.0)	3 (18.8)	6 (33.3)	11 (15.5)
Malaria/dengue	0 (0)	0 (0)	0 (0)	0 (0)	0 (0)
Embolicdisease (thrombo/amniotic/air embolism)	0 (0)	0 (0)	0 (0)	0 (0)	0 (0)
Cancer	0 (0)	0 (0)	1 (6.3)	0 (0)	1 (1.4)
Heart disease	0 (0)	0 (0)	2 (12.5)	1 (5.6)	3 (4.2)
Lung disease	0 (0)	0 (0)	0 (0)	0 (0)	0 (0)
Renal disease	1 (3.1)	1 (20.0)	1 (6.3)	1 (5.6)	4 (5.6)
Hepatic disease	1 (3.1)	2 (40.0)	0 (0)	0 (0)	3 (4.2)
Coincidental conditions (includes violence, accident, poisoning, self-harm)	0 (0)	0 (0)	0 (0)	0 (0)	0 (0)

Table 5 presents the types of organ dysfunctions experienced by nearmiss women. At RHUH in Lebanon, the most frequently reported dysfunction among maternal near-miss cases was hepatic dysfunction (80 % of the cases). At Al-Galaa hospital in Egypt, a high frequency of cardiovascular dysfunction was noted (59.4 % of the cases). At Dar Al Tawleed hospital in Syria, coagulation dysfunction was the most frequently reported dysfunction among maternal near-miss cases (83.3 %). Overall, coagulation dysfunctions among maternal near-miss cases were the most prevalent type of dysfunction, followed by cardiovascular dysfunctions.

**Table 5:** Frequency of organ dysfunction among maternal near-misscases in the four study hospitals^a,b^

	Egypt	Lebanon	Palestine	Syria	All
	Al Galaa Hospital	Rafik Hariri University Hospital	Ramallah Hospital	Dar Al Tawleed Hospital	
	*N* = 32	*N* = 5	*N* = 16	*N* = 18	*N* = 71
	N (%)	N (%)	N (%)	N (%)	N (%)
Cardiovascular dysfunction	19 (59.4)	0 (0)	2 (12.5)	3 (16.7)	24 (33.8)
Respiratory dysfunction	2 (6.3)	0 (0)	2 (12.5)	5 (27.8)	9 (12.7)
Renal dysfunction	1 (3.1)	3 (60.0)	2 (12.5)	2 (11.1)	8 (11.3)
Coagulation dysfunction	24 (75.0)	3 (60.0)	12 (75.0)	15 (83.3)	54 (76.1)
Hepatic dysfunction	4 (12.5)	4 (80.0)	0 (0)	0 (0)	8 (11.3)
Neurologic dysfunction	2 (6.3)	1 (20.0)	2 (12.5)	0 (0)	5 (7.0)
Uterine dysfunction	7 (21.9)	2 (40.0)	4 (25.0)	6 (33.3)	19 (26.8)

^a^For description of organ dysfunction, please note reference 21

^b^Some cases may have encountered more than one dysfunction

Table 6 shows the coverage of selected key clinical interventions implemented for the near-miss cases in the four hospitals during the data collection period. It is clear that the coverage of these clinical interventions was either universal or very high in the study hospitals. Overall, in 100 % of the eclampsia cases magnesium sulphate was used. Prophylactic antibiotics were administered to 90.9 % of all the cesarean cases; Al-Galaa hospital in Egypt had the lowest coverage, at 83.2 %. Among all delivering women at the study hospitals, 86.6 % received prophylactic oxytocin as a preventative measure against postpartum haemorrhage. Regarding therapeutic oxytocin, a necessary intervention for cases of postpartum haemorrhage, its coverage among near-miss cases was nearly universal across the four contexts. Coverage of blood products (administering any blood products, i.e. blood, red cells, plasma, platelets) among maternal near-miss cases with postpartum haemorrhage was 100 %. Laporatomy was performed in 50 % of all near-miss cases with uterine rupture. No cases of sepsis were reported among the maternal near-miss cases.

**Table 6:** Coverage of key clinical interventions in the four study hospitals

	Egypt	Lebanon	Palestine	Syria	All
	Al Galaa Hospital	Rafik Hariri University Hospital	Ramallah Hospital	Dar Al Tawleed Hospital	
	N (%)	N (%)	N (%)	N (%)	N (%)
Coverage of prophylactic oxytocin among all who delivered at hospital	1921/2736(70.2)	1171/1178 (99.4)	1119/1234 (90.7)	3967/4007 (99)	8178/9423 (86.8 )
Coverage of therapeutic oxytocin among near-miss cases with postpartum haemorrhage	3/3 (100)	1/1 (100)	5/6 (83.3)	4/4 (100)	13/14 (92.9)
Coverage of blood products among near-miss cases with postpartum haemorrhage	3/3 (100)	1/1 (100)	6/6 (100)	4/4 (100)	14/14 (100)
Coverage of magnesium sulphate among near-miss cases with eclampsia	2/2 (100)	1/1 (100)	1/1 (100)	1/1 (100)	5/5 (100)
Coverage with any other anticonvulsant among near-miss cases with eclampsia	0/2 (0)	1/1 (100)	1/1 (100)	N/A	2/4 (50)
Coverage of prophylactic antibiotics among women with caesarean section	1238/1488 (83.2)	427/432 (98.8)	275/307 (89.6)	913/913 (100)	2853/3140 (90.9)
Coverage of parenteral antibiotics among maternal misses with sepsis^a^	N/A	N/A	N/A	N/A	N/A
Coverage with laparotomy among near-miss cases with uterine rupture	1/4 (25)	N/A	1/1 (100)	2/2 (100)	4/8 (50)

^a^No sepsis cases were reported

## Discussion

This is the first time maternal near-miss cases in public hospitals are systematically and comparatively reported in four Middle Eastern, middle income developing countries, namely Egypt, Lebanon, Palestine and Syria. The use of the WHO multicountry data collection form was advantageous for comparative purposes within the region and to other regions. Though hospitals from two countries (Lebanon and Palestine) took part in the global WHO MCS study but findings from the Middle East region have been presented on its own [21]. A previous study in Syria reported maternal near miss using other criteria [22], and a separate study was conducted in Iraq [9].However, the systematic and collaborative nature of our work facilitated in-depth comparison specific to the context of the region. It also laid the basis for the design of a quality improvement intervention as part of the study using clinical audit and feedback on the near-miss cases (to be reported separately).

During the study period, overall 0.85 % of eligible women admitted to the four study hospitals developed a severe maternal morbidity. This is slightly lower than that reported by Souza (1 %) in the WHO multicountry study that included 29 countries; among them low-income and high maternal mortality contexts [21]. The fact that our study was carried out in middle income countries with high levels of facility-based births and access to obstetric care may explain this.

The overall maternal near-miss ratio for the four contexts was 7.8/1,000 live births, which is slightly lower than the overall ratio reported by Souza et al. in 2013 (8.3/1000 live births) [21]. In the WHO MCS study, the countries are presented by categories according to maternal mortality ratio (MMR); three out of the four Middle Eastern countries included in the WHO MCS are categorized as having a moderate MMR (Jordan, Lebanon and Palestine), and only one (Qatar) as a country with alow MMR. Our study’smaternal near miss ratio (7.8/1000 live births) was found higher when compared to the maternal near miss ratio (6.1/1000 live births) of those countries categorized by WHO MCS study as moderate MMR. The higher severe maternal outcome ratio (SMOR) in both the Egyptian (13.3 per 1000 live births) and the Palestinian (12.9 per 1000 live births) hospitals suggest that the amount of care and resources that would be needed in both facilities is high [20].The overall SMOR for the WHO MCS countries reported in Souza el al (2013) was 9.9/1,000 live births and in countries with a moderate MMR the SMOR was 6.5/1,000 live births [21]. The overall maternal mortality index in our study was 7.8 % with higher indices in both the Egyptian and Syrian hospitals. The overall maternal mortality index in the WHO MCS study was 16.1 % and for the countries in that study with a moderate MMR, the mortality index was recorded at 5.6 % [21]. Jabir’s study in urban Iraq reported a mortality index of 11.1 % [9]. A higher index reflects that more women with life-threatening conditions die (i.e., lower quality of care) [20]. Therefore, the fact that the study hospitals had a higher mortality index indicates possibly lower quality of care. The fact that both hospitals are the main referral hospitals with a wide case mix in these large urban settings may explain the higher mortality.It is also worth mentioning that some poor outcomes may have been due to delay in treatment, but timing was not measured in this study.Further in-depth investigation is needed to identify whether lack of timeliness of interventions and treatments might have been detrimental to maternal health outcomes.

It was noted that postpartum haemorrhage was the main contributing factor to severe maternal outcomes in the study hospitals as compared to infection which was not reported at all in our data. Souza and colleagues [21] reported lower proportions of postpartum haemorrhage and higher proportions of infection as compared to our findings. Postpartum haemorrhage was also found to be the major contributor to maternal mortality in Syria and Egypt in nation-wide population studies [17–19]. Of the indirect causes of severe maternal outcomes, anemia was the most common in our countries. This was noted in the WHO MCS study as well as in other studies in developed countries [21, 23].

Of the maternal characteristics that were correlated with the severity of maternal outcomes, age and education were noted. Women who experienced a near miss event tended to be older in both Palestine and Syria and were less educated in Lebanon as compared to women not classified as near-miss cases. Higher proportions of the near-miss cases had a history of previous cesarean sections in Lebanon and Egypt as compared to other contexts, which is significant given that Lebanon has a much higher c-section rate nationally compared to the other countries.These findings are consistent with the findings from the WHO multicountry study [21, 24, 25]. The c-section rate in these four study contexts ranges from a high of 46 % nationally in Lebanon [14] and 27.6 % in Egypt [26], to 20.3 % in Palestine [27] and 13.6 % in Syria before the current conflict in the country [28]. Importantly, the main contributing conditions to maternal near miss in RHUH, in Lebanon and Al Galaa, in Egypt were abnormal placentation (including placenta previa, accreta/percreta&abruptio). Internationally, studies have documented the association between the increased incidence of placenta previa and accreta with the increase in cesarean delivery rates and in the number of previous c-sections [29, 30]. These findings are very relevant and point to the importance of exploring the negative impact of high population c-section rates on maternal health. Indeed, in Lebanon, obstetrician-gynecologists have expressed concern about the higher level of placental conditions being observed clinically following repeat c-sections (Abu Seoud, personal communication).

Our study had similar findings to the WHO MCS study in that a high reported level of coverage of key evidence-based interventions co-existed with considerable disparities in maternal health outcomes. As noted, our study found a higher prevalence of severe maternal health outcomes as compared to countries with a moderate MMR in the WHO MCS [21]. This points to the need to address wider quality of care issues within the health system contexts of these settings and further investigate the implementation of clinical protocols as well as the timely applications of specific interventions.

Our study has certain limitations. The main one is the fact that the number of hospitals in our study is few and that they are all large referral hospitals which means that the study’s findings cannot be said to be representative of the study contexts. Moreover, in the case of Palestine and Lebanon the case load was relatively low. Furthermore, it was not possible to obtain data on all eligible women in Egypt due to the large load of deliveries in that hospital and the resulting demands for data collection. There were missing data on maternal characteristics in Egypt and Palestine as this information was not included in the medical records in those settings. This hampered the ability to compare the findings and to do multivariate analysis to highlight the main risk factors of severity. As in the WHO multi-country survey, data was collected up to the seventh postpartum day, even though the definition of a maternal near miss is based on a duration of 42 days after pregnancy termination. This was done to ensure proper and feasible data collection with a limited burden to the data collectors. Hence, some women who have experienced a maternal near miss event in our settings may not have survived, or may have had further complications during the remaining pastpartum period. On the other hand, our study had several strengths. The research teams including the hospital staff in the four contexts gained experience in collecting data on severe maternal and neonatal outcomes and this was used to assess the impact of the intervention on the management of maternal near-miss casesthat will be reported elsewhere. Use of the internationally validated WHO tool was also tested in the contexts of these four settings during the formative stage which informed the revision of the WHO tool and the inclusion of context-relevant factors for the intervention stage.The standard tool as well as the standard operational definitions as per WHO manual [20] might have improved the quality of the work.

## Conclusions

The findings of this research concerning the higher mortality index and the lower maternal near miss mortality ratio as compared to the indicators in the countries with a moderate MMR in the WHO multicountry study suggest poorer quality of care in the study hospitals. Furthermore, the high reported coverage of the main clinical interventions in the study hospitals would appear to be in contradiction with the above findings. This indeed justifies our assumption that the quality of care issues need to be tackled in the setting of the four hospitals and other similar ones in our region.

## Additional files

Additional file 1:
**Data collection tool - WHO individual form (HRP A65661).** (DOCX 308 kb) Additional file 2:
**Data collection tool - WHO individual form (HRP A65661) – Translated into Arabic.** (DOCX 75 kb)

## Abbreviations

CCCC: choices and challenges in changing childbirth
MCS: multicountry study
MMR: maternal mortality ratio
MNM: maternal near miss
NGOs: Non-Governmental Organizations
UNRWA: United Nations Relief and Works Agency
WHO: World Health Organization

## Footnotes

1 The Choices and Challenges in Changing Childbirth Regional Network’s website: http://www.aub.edu.lb/fhs/cccc/Pages/index.aspx

2 www.csprousers.org
