# Supplementary figures and images for: A cross sectional study of maternal ‘near-miss’ cases in major public hospitals in Egypt, Lebanon, Palestine and Syria
Source: BMC Pregnancy Childbirth. 2015 Nov 13;15:296. doi: 10.1186/s12884-015-0733-7 (PMC4644334; doi:10.1186/s12884-015-0733-7)

**Additional file 1:**Data collection tool - WHO individual form (HRP A65661)


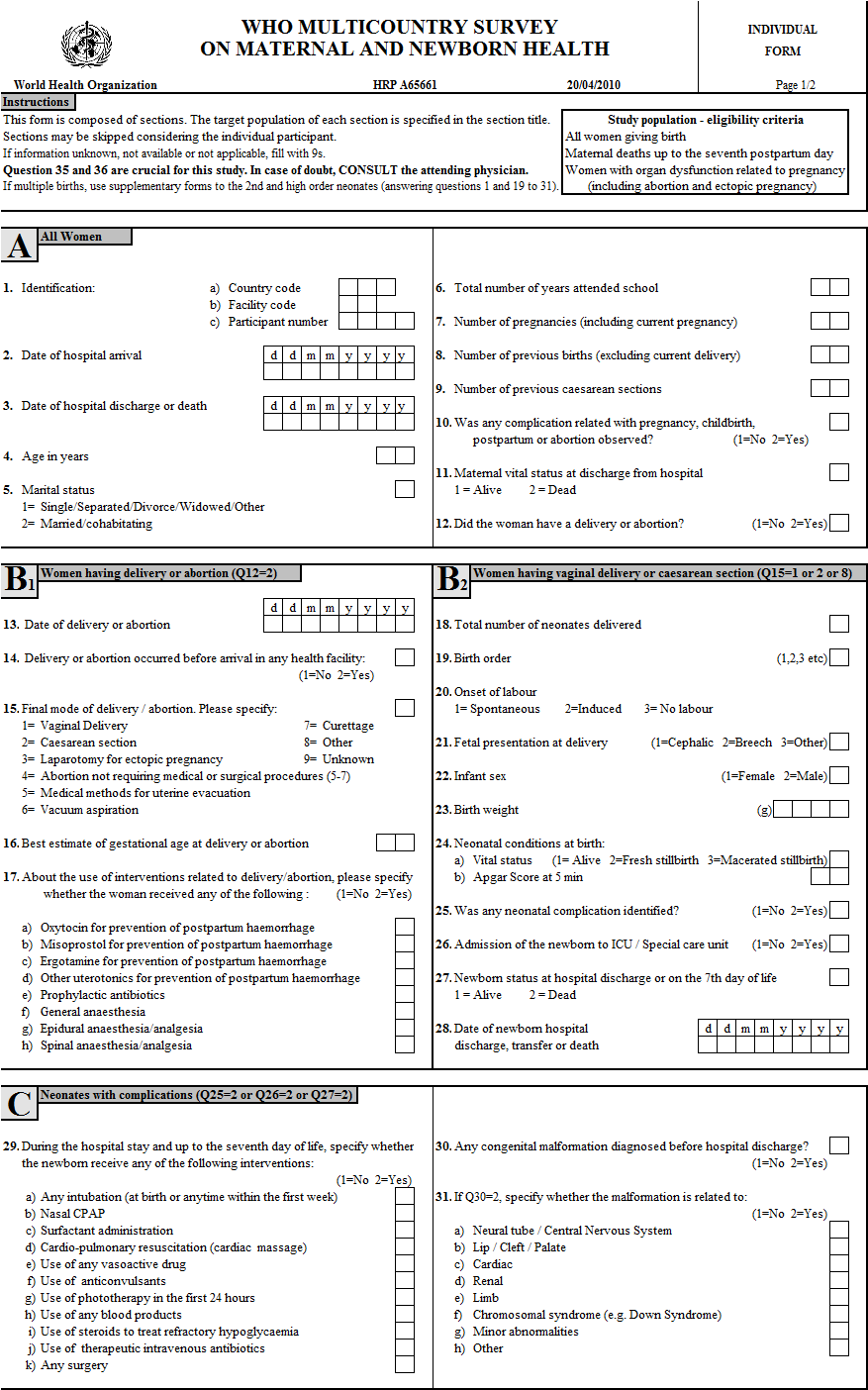


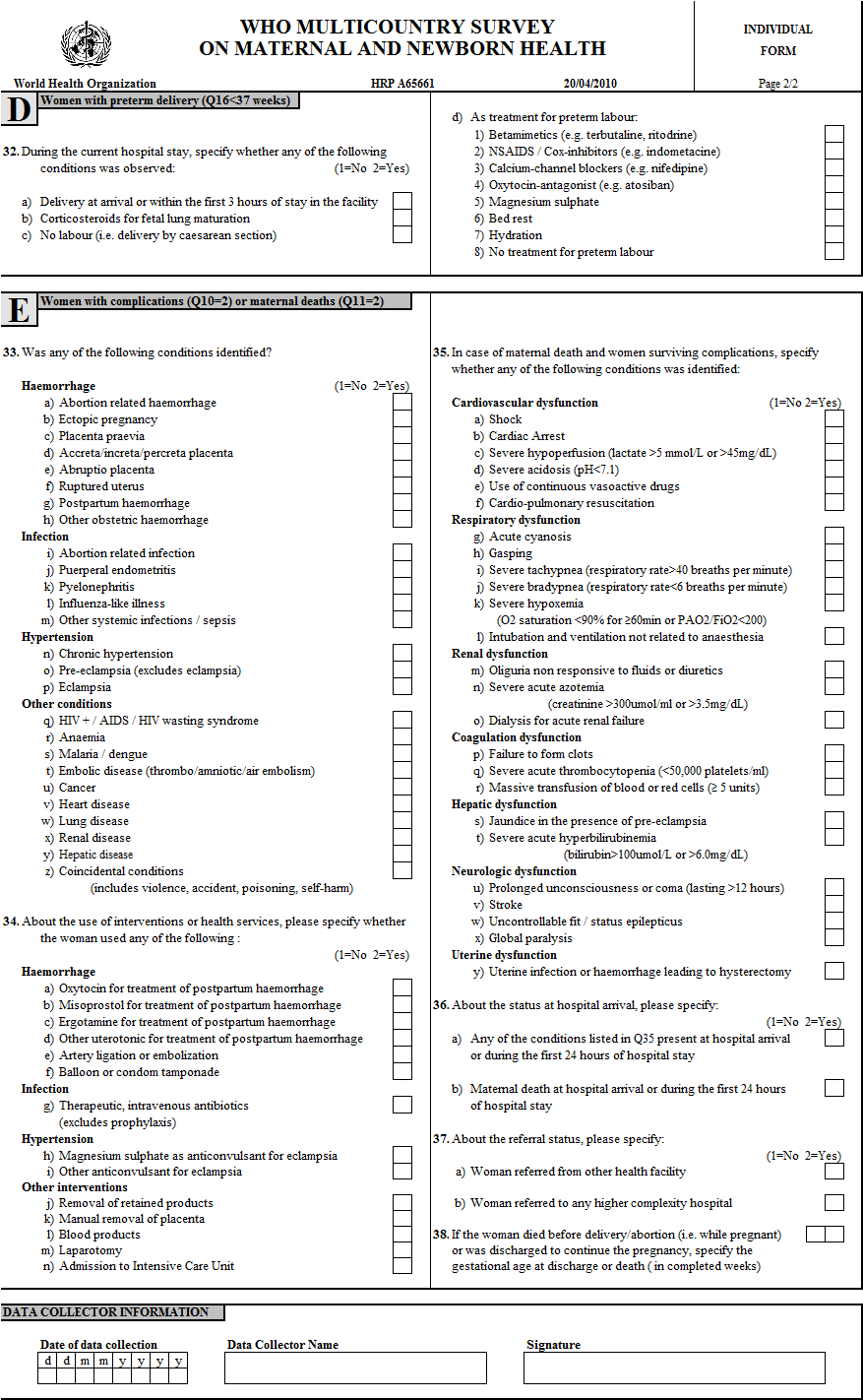

Supplement: Additional file 1: — Data collection tool - WHO individual form (HRP A65661). (DOCX 308 kb) [file 12884_2015_733_MOESM1_ESM.docx]
